# Supplementary material for: Definitions of Incidental [18F]FDG PET/CT Findings in the Literature: A Systematic Review and Definition Proposal
Source: Diagnostics (Basel). 2024 Dec 9;14(23):2764. doi: 10.3390/diagnostics14232764 (PMC11639948; doi:10.3390/diagnostics14232764)
Supplement: Supplementary file 1 [file diagnostics-14-02764-s001.zip › diagnostics-3313649-supplementary.pdf]

Supplemental Table S1. Overview of IPF definitions in the included studies and their compliance with the *Wording 1* and *2* criteria.

Organ site, author	Definition of IPF	Wording 1 criteria fulfilled	Wording 2 criteria fulfilled
Thyroid, Achury et al. [25]	"A thyroid incidentaloma is defined as a focal or diffuse increase in the glandular uptake of FDG incidentally detected in a patient studied for a non-thyroidal pathology"	1, 2	1, 2
Thyroid, Bahl et. al. [44]	"An incidentaloma was defined as a finding that was originally detected on imaging studies performed for other reasons in patients without any clinical symptoms, examination findings, or suspicion for thyroid cancer"	4	1, 2, 5
Thyroid, Barrio et. al. [36]	"Thyroid incidentalomas, defined as thyroid lesions identified during an imaging study such as a computed tomography (CT), magnetic resonance imaging (MRI) or PET/CT studies for evaluating non-thyroid disease"	4	1, 2
Thyroid, Bertagna et. al. [60]	"Thyroid incidental uptake is defined as a thyroid uptake incidentally and newly detected by imaging techniques performed for an unrelated purpose and especially for non-hyoid diseases"	1	1, 2, 4
Thyroid, Bertagna et al. [26]	"Thyroid incidentaloma is commonly defined as a thyroid uptake incidentally and newly detected by imaging techniques performed for an unrelated and non-thyroid purpose"	1	1, 2, 4

Thyroid,<br>Choi et. al. [23]	"A thyroid incidentaloma was defined as a focal area of the thyroid showing uptake on 18F-FDG PET images. Focal uptake of the thyroid was defined as 18F-FDG uptake occupying less than one lobe"	1, 2	*
Thyroid,<br>Chun et. al. [17]	"Thyroid incidentaloma was defined as focal thyroid uptake identified incidentally on 18F-FDG PET/CT study"	1, 2	1
Thyroid,<br>Demir et. al. [57]	"Thyroid incidentaloma was defined as thyroid uptake identified on 18F-FDG PET study incidentally and was divided into focal and diffuse types according to the thyroid uptake pattern of 18F-FDG"	1, 2	1
Thyroid,<br>Gavriel et. al. [41]	"Thyroid incidentaloma was defined as thyroid uptake identified on FDG-PET study incidentally, and it was divided into focal and diffuse types according to the thyroid uptake pattern of FDG"	1, 2	1
Thyroid,<br>Kao et al. [50]	"A FDG-avid thyroid incidentaloma was defined as focal unilateral thyroid FDG uptake higher than the background thyroid bed and surrounding blood pool by visual assessment, regardless of any underlying lesions seen on CT"	1, 2, 3	*
Thyroid,<br>Kim et al. [35]	"Thyroid incidentalomas are defined as newly detected thyroid lesions on an imaging study for non-thyroid disease"	4	1, 2, 4
Thyroid,<br>Larg et al. [24]	"An unexpected and asymptomatic thyroid nodule discovered accidentally during imaging studies or a surgical intervention performed for unrelated pathologies of the thyroid"	4	1, 2, 5

Thyroid,<br>Law et al. [45]	"Incidental thyroid tumor is defined as incidentally identified by imaging study performed for reason(s) unrelated to the thyroid gland"	4	1, 2
Thyroid,<br>Lee et al. [51]	"Thyroid incidentaloma was defined as a newly identified thyroid lesion on PET or PET/CT in a patient without a previous, known history of thyroid disease"	4	3, 4
Thyroid,<br>Nilsson et al. [48]	"A thyroid incidentaloma is defined as a mass or lesion detected incidentally by an imaging modality performed for reasons that are not related to clinical suspicion of a thyroid disorder"	4	1, 5
Thyroid,<br>Qu et al. [31]	"Thyroid incidentalomas, defined as focal thyroid lesions encountered during imaging with PET/CT"	1, 2	*
Thyroid,<br>Soelberg et al. [32]	"Newly identified focal thyroid lesions encountered during imaging with PET/CT"	1, 2	4
Thyroid,<br>Thuillier et al. [40]	"TI was defined as the presence of a focal FDG thyroid uptake (single or multiple) on FDG PET–CT higher than the adjacent thyroid parenchyma uptake"	1, 2, 3	*
Thyroid,<br>Treglia et al. [28]	"Thyroid incidentalomas are defined as unexpected, asymptomatic thyroid lesions that are discovered on an imaging study or during surgery unrelated to the thyroid"	4	1, 2, 5
Thyroid,<br>Yoon et al. [39]	"Thyroid ''incidentalomas'' are defined as focal thyroid lesions detected on various imaging modalities performed for the purposes of evaluating other regions of the neck"	4	1

Thyroid,<br>Kamakshi et al. [64]	"A thyroid incidentaloma, by definition, is any clinically evident thyroid nodule picked up during imaging done for the evaluation of a different disease process"	4	1, 2
Thyroid,<br>Familiar et al. [75]	"Thyroid incidentaloma was defined as either focal (in less than one lobe) or diffuse (uptake in the whole gland) FDG uptake within the thyroid gland in subjects without a previous diagnosis of thyroid cancer"	1, 2	3
Thyroid,<br>Mcqueen et al. [73]	"Thyroid incidentaloma can be defined as an asymptomatic and unexpected thyroid lesion identified incidentally on imaging performed to investigate conditions unrelated to the thyroid itself"	4	1, 2, 5
Thyroid,<br>Wadsley et al. [72]	"Thyroid incidentaloma are defined as focal uptake greater than the background thyroid. Focal thyroid uptake which is greater than that of normal liver parenchyma, mediastinal blood pool or two to three times greater the background thyroid"	1, 2, 3	*
Breast,<br>Bertagna et al. [6]	"Breast incidentaloma could be defined as a breast lesion incidentally and newly detected by imaging techniques performed for an unrelated purpose and especially not for breast diseases"	4	1, 2, 4
Breast,<br>Shin et al. [47]	"Incidental focal FDG uptake in the breast was defined as a focally increased FDG accumulation in the breast a woman without a history of breast cancer"	1, 2	3
Prostate,<br>Bertagna et. al. [59]	"Prostate incidental uptake (PIU) is defined as incidental uptake by the prostate which can be detected by PET/CT performed for an unrelated purpose, especially non-prostate disease"	4	1, 2

Prostate,<br>Yang et al. [27]	"Incidental FDG uptake was defined as discrete FDG activity higher than the surrounding prostate gland on visual analysis"	1, 3	*
Prostate,<br>Hwang et. al. [49]	"Definition of abnormal hypermetabolism in the prostate gland: Focal hypermetabolism was considered as significant if it was evident as an asymmetrical placement on one side of the prostate gland"	1, 2, 3	*
Prostate,<br>Cho et al. [37]	"A focal prostate lesion was defined as focally increased 18F-FDG uptake within the prostate glands compared with the uptake of surrounding prostatic parenchyma, excluding the focal physiological uptake in the prostatic urethra which was discernible by fused PET/CT images"	1, 2, 3	*
Prostate,<br>Kang et al. [18]	"Hypermetabolism of the prostate gland was defined as discrete FDG activity higher than that of the surrounding prostate gland on visual analysis"	1, 3	*
Prostate,<br>Mannas et al. [66]	"Incidental focal uptake in any organ on FDG PET that is not directly associated with the disease under investigation is referred to as an incidentaloma"	1, 2	1, 2
Parotid,<br>Bothe et. al. [46]	"Parotid gland incidentalomas (PGIs) are defined as new focal intraglandular deposits of radiotracer in patients without prior history of parotid disease"	1, 2	3, 4
Parotid,<br>Thompson et al. [67]	"Unexpected, asymptomatic tumors discovered serendipitously during an unrelated procedure"	4	1, 5

Parotid,<br>Treglia et al. [5]	"Parotid incidentalomas are defined as unexpected, asymptomatic lesions in the parotid gland that are discovered on an imaging study performed for reasons unrelated to the parotid gland"	4	1, 2, 5
Salivary glands<br>Danstrup et al. [71]	"Incidental salivary lesions defined as a focal FDG-positive lesion in patients without a prior history of salivary gland diseases"	1, 2	3
Gastrointestinal,<br>Garrido et al. [29]	"Incidental uptake was defined as the focal accumulation of 18F-FDG in patients that underwent a pathological study that was not related to the colon or in areas incompatible with the previously known pathology"	1, 2	1, 2
Gastrointestinal,<br>Mui et al. [61]	"Focal colorectal FDG uptake is termed incidental if it is detected in the large bowel on PET/CT in patients who have a non-colorectal or unknown primary cancer"	1, 2	1, 3
Gastrointestinal,<br>Salazar et al. [33]	"Incidental FDG accumulation was defined as focal, segmental or multifocal colorectal FDG uptake that was unrelated to the usual site of metastasis of a known tumor, or was not associated with a known benign disease, or was not related to physiological elimination of the radionuclide"	1, 2	6
Gastrointestinal,<br>Servente et al. [42]	"The colorectal incidentaloma is defined as the unexpected finding of a colorectal focal uptake in the PET/CT scan of a patient with non-colon cancer"	1, 2	1, 3
Gastrointestinal,<br>Shmidt et al. [22]	"An incidental finding was defined as an area of FDG uptake in the GIT that could not be expected based on the patient's known medical history at the time of the PET/CT scan"	1	3

Gastrointestinal, Treglia et al. [43]	"Colorectal uptake of FDG was termed as incidental if detected in the colon/rectum of a patient without a known colorectal disease, or if detected in a bowel location distinct from that of the colorectal primary tumour"	1	2, 3, 6
Gastrointestinal, Treglia et al. [52]	"Colorectal incidentalomas (Cis) are defined as unexpected colorectal findings that are discovered on an imaging study unrelated to the large bowel"	4	1, 2
Gastrointestinal, Valente et al. [38]	"Incidental uptake was defined as focal activity in the colon or rectum in patients undergoing PET/CT scan for a non-colorectal disease process, regardless of SUVmax uptake"	1	2
Gastrointestinal, Young et al. [21]	"Incidental colorectal lesions were defined, as in previous studies, as 1) FDG-avid lesions identified in a location that would not be an expected endpoint for a particular primary malignancy, 2) persistent activity in a distant site when the primary tumor or similar lesions had resolved or become significantly less FDG-avid, or 3) incidental findings in sites not expected on the basis of the reason for ordering the PET/CT"	1	1, 6
Gastrointestinal, Hui et al. [68]	"Incidental focal colonic FDG uptake was defined as focal accumulation of activity greater than the surrounding background in the colon that could not be explained based on the patient's known medical history at the time of scanning"	1, 2, 3	3, 4

Gastrointestinal,<br>Zhang et al. [69]	"An incidental finding was defined as an unexpected area of elevated FDG uptake in the ascending colon, transverse colon, descending colon, sigmoid colon, or rectum"	1	*
Pituitary,<br>Hyun et al. [55]	"Focal 18F-FDG accumulation in the pituitary gland was defined as increased 18F-FDG uptake localized in the sellar area that was greater than background activity in adjacent tissues"	1, 2, 3	*
Pharynx and Larynx,<br>Al-Hakami et al. [15]	"A new head and neck abnormal uptake, focal or diffuse, identified on FDG-PET study incidentally in a patient without a prior history of head and neck disease"	1, 2	1, 3, 4
Pharynx,<br>Armstrong et al. [74]	"Incidental asymmetric uptake was defined as asymmetry between the right and left sides of the oropharynx in patients with no history or pre-test suspicion of oropharyngeal cancer"	1	3
Gynecological,<br>Bellini et al. [62]	"An asymptomatic and unexpected lesion found during the course of different and heterogeneous imaging examinations performed for unrelated purpose, usually involving a different organ"	4	1, 2, 5
Adrenal,<br>Kebebew et al. [65]	"An adrenal incidentaloma is defined as a clinically unapparent adrenal lesion (≥1 cm in diameter) that is detected on imaging performed for indications other than evaluation for adrenal disease"	4	2
Multiple organs,<br>Chopra et al. [30]	"An incidental finding was defined as either a significant area of FDG uptake at a site where the uptake was unlikely to be related to the primary neoplasm, but could represent significant pathology, or an abnormality on the CT component of the PET/CT scan that was not evident on the initial diagnostic CT scan"	1	1, 6

Multiple organs, Gill et al. [19]	"Incidental intraabdominal FDG-uptake was defined as "focal" and intense uptake compared to background activity as observed by the nuclear medicine physicians"	1, 2, 3	*
Multiple organs, Adams et al. [20]	"An incidental finding was defined as any focal accumulation of FDG thought to be unrelated to the primary malignancy. This decision was based on the incidental finding having an atypical location, intensity or pattern for metastasis"	1, 2	1, 6
Multiple organs, Tae et al. [54]	"Incidental finding on PET/CT was defined as any finding not related to gastric cancer that was detected by the radiologist who interpreted the studies"	4	1
Multiple organs, Moletta et al. [58]	"An incidental finding was defined as a significant area of FDG uptake in a site unlikely to be related to the pancreatic neoplasm"	1	1, 6
Multiple organs, Shabbir et al. [53]	"Clinically significant incidental finding (CSIF) is defined as any finding that was new and needed further investigation or follow up"	4	4, 7
Multiple organs, Wan et al. [34]	"Incidental findings, the outcome of interest, were defined as observations of potential clinical significance that were discovered and unrelated to the purpose or beyond the aims of the research studies in healthy, asymptomatic subjects or symptomatic patients with seemingly unsuspecting symptoms"	4	1, 5, 7
Multiple organs, Husmann et al. [63]	"Findings not previously known and considered to harbor potential impact on patient management, they were defined as "unknown incidental findings"	4	3, 7

Multiple organs, Adams et al. [11]	"IFs were defined as either a significant area of hypermetabolism or hypometabolism, or an abnormality on the CT component of the PET/CT scan considered unrelated to the clinical indication for imaging as provided by the referring physician"	1	1, 6
Multiple organs, Schaaf et al. [16]	"Unexpected findings that are discovered when imaging is ordered for a completely unrelated cause"	4	1
Multiple organs, Sheldon et al. [56]	"Defined as any unknown or unexpected abnormality detected, other than the known primary pathology as documented by the referring clinician"	4	*
Multiple organs, Tamburello et al. [4]	"Asymptomatic and unexpected lesions, found during the course of examination and imaging for other reasons and related to a different organ"	4	1, 2, 5
Multiple organs, Hans et al. [70]	"An incidental finding was defined as an area of FDG uptake on the PET component or an abnormality on the CT component that was unlikely to be related to large vessel vasculitis and had not been documented in previous imaging studies"	1	1, 4

* no criteria found
